# A Long-Term Experimental Study Demonstrates the Costs of Begging That Were Not Found over the Short Term

**DOI:** 10.1371/journal.pone.0111929

**Published:** 2014-11-05

**Authors:** Manuel Soler, Francisco Ruiz-Raya, Laura G. Carra, Eloy Medina-Molina, Juan Diego Ibáñez-Álamo, David Martín-Gálvez

**Affiliations:** 1 Departamento de Biología Animal, Facultad de Ciencias, Universidad de Granada, Granada, Spain; 2 Grupo Coevolución, Unidad Asociada al CSIC, Universidad de Granada, Spain; 3 Departamento de Ecología Funcional y Evolutiva, Estación Experimental de Zonas Áridas, Almería, Spain; University of Lausanne, Switzerland

## Abstract

Parent–offspring conflict theory predicts that begging behaviour could escalate continuously over evolutionary time if it is not prevented by costliness of begging displays. Three main potential physiological costs have been proposed: growth, immunological and metabolic costs. However, empirical evidence on this subject remains elusive because published results are often contradictory. In this study, we test for the existence of these three potential physiological costs of begging in house sparrow (*Passer domesticus*) nestlings by stimulating a group of nestlings to beg for longer and another group for shorter periods than in natural conditions. All nestlings were fed with the same quantity of food. Our study involves a long-term experimental treatment for begging studies (five consecutive days). Long-term studies frequently provide clearer results than short-term studies and, sometimes, relevant information not reported by the latter ones. Our long-term experiment shows (i) a clear effect on the immune response even since the first measurement (6 hours), but it was higher during the second (long-term) than during the first (short-term) test; (ii) evidence of a growth cost of begging in house sparrow nestlings not previously found by other studies; (iii) body condition was affected by our experimental manipulation only after 48 hour; (iv) a metabolic cost of begging never previously shown in any species, and (v) for the first time, it has shown a simultaneous effect of the three potential physiological costs of begging: immunocompetence, growth, and metabolism. This implies first, that a multilevel trade-off can occur between begging and all physiological costs and, second, that a lack of support in a short-term experiment for the existence of a tested cost of begging does not mean absence of that cost, because it can be found in a long-term experiment.

## Introduction

Communication drives most of the interactions between individuals in the natural world, including animals, plants, and microorganisms [1], [2]. Among the different types of communication, that occurring between parents and offspring has been a central issue of communication theory. Since the publication of sibling scramble competition models and, especially, honest signaling models ([3]–[6]; see below) intense empirical research has been performed and many experimental papers on the topic have been published, giving rise to important theoretical advances in communication theory [7].

In species with parental care, parents are selected to optimize their investment in parental care in such a way that maximizes the translation of provided resources into offspring fitness [8]. This important selective pressure favors the evolution of parent-offspring communication, in which offspring demand care by producing signals (visual, acoustic, chemical or tactile) and parents allocate their investment according to those signals [7].

Altricial birds have been the most commonly used model species in the study of parent-offspring communication [7]. Begging signals by altricial nestlings usually involve vigorous and exaggerated displays, which include brightly colored gapes, neck stretching, wing flapping, and noisy calls [7]. Such exuberant begging behaviour is considered to be the evolutionary outcome of a genetic conflict of interests within the family over resource allocation between parents and offspring [3]–[5] and among offspring themselves [3], [6]. In the first case, it is assumed that the conflict arises because nestlings are selected to demand a larger share of investment than parents would be selected to provide, since it would compromise their future fitness [9]. In the second case, competition among nestlings could also drive the evolution of exaggerated begging behaviour under conditions of limited parental resources [5], [10], [11].

The two above-mentioned conflicts of interests regarding resource allocation explain the existence of vigorous begging displays in altricial nestlings, but they further predict that begging behaviour could escalate continuously over evolutionary time if not prevented by costliness of begging signals. Both sibling-scramble competition and honest-signaling models predict that the aforementioned conflicts of interests could be solved only if begging signals are costly to produce. These costs, by the higher increase of marginal costs compared to benefits of begging production, would constrain the expression of offspring solicitation signals, limiting the escalation of sibling competition and enforcing honest signaling, thus allowing an optimal level of begging, which would lead to a stable equilibrium (for reviews, see [12]–[16]; but see [17]–[21] for other explanations considering that begging signals do not necessarily have to be costly). Given that begging should be costly in order to be evolutionarily stable, it is crucial to know the costs associated with begging signals in order to understand the evolution of begging behaviour. During the last 25 years, many empirical studies have been performed trying to determine such costs. However, whether begging behaviour really implies fitness costs remains controversial [22]–[25] because published results are scarce and often contradictory.

Three types of begging costs that could contribute to avoid the escalation of begging signals have been proposed: an indirect cost provoked by a reduction in inclusive fitness [19], [26]–[28], costs related to increased predation risk [29]–[36] and physiological costs that would be directly related to the intensity of the begging displays.

Several potential physiological costs have been proposed. Energy expenditure during begging was found to be only slightly higher than the resting metabolic rate [22], [37]–[41], while mass loss triggered by begging activity resulted marginally different between treatments (nestlings forced to beg hard vs. nestlings begging at a low rate [42], or very similar for both experimental groups of nestlings [43]. Thus, both approaches found that the energy cost of begging is low.

The existence of growth costs has been tested in six species, but results have been contradictory. No significant reduction in growth in relation to experimentally increased begging activity has been reported in three of them [24], [43]–[45], but growth costs associated with begging have been reported in the other three species [42], [45]–[47].

Another potential physiological cost associated with begging is a reduction of the cell-mediated immune response [25], [48]–[51], which is an important defense against pathogens. Mounting an immune response as well as the development of the immune system is expensive [49], [52]–[54], and thus an excessive cost of begging could provoke a cost in terms of immunocompetence. This would imply an important begging cost because lower immunocompetence in nestlings begging dishonestly would jeopardize their resistance to infections and it is well known that nestlings with reduced immune capacity have a higher mortality risk [55]–[57]. An immunological cost of begging have been clearly documented given that it has been experimentally demonstrated in the three species hitherto tested [43], [47], [58].

Although the energetic expenditure of begging is small (see above), growth and development of altricial nestlings involve many highly demanding energy processes that compete for resources [59]. Rapid growth is selected for by the risk of nest predation [60], [61], but growth rates might be constrained by physiological factors other than immune response (see above), which would prevent nestlings from growing faster [60], [62]–[64]. Thus, given that nestlings allocate to growth only 13–28% of their total metabolized energy [65], a small increase in energy expenditure for begging could lead to relatively high begging costs [38], [65]. In this scenario, an excessive investment in begging would probably influence a nestling's distribution of the total energy budget among different fitness traits, which could in turn affect the phenotypes and survival prospects of developing nestlings. In fact, aside from the effect of begging on several traits commented above, several studies have demonstrated a trade-off between growth and immune response ([66]–[68]; but see [43], [58] and discussion below) and it has been shown that ecological conditions may affect priority rules in the allocation of resources between the two fitness traits [69].

Long-term studies provide indispensable information, which cannot be reported by short-term studies, not only in evolutionary ecology, but also in science in general [70]–[73]. Furthermore, long-term studies can show clearer results orders of magnitude higher than those shown by short-term studies [62], [74] and are highly recommended [75], [76]. On the other hand, another key issue in science is replication of experimental studies, because it is important to eliminate the possibility that a significant result could appear just by chance [77], [78].

Here, we perform a long-term experiment (five days of experimental manipulation) in order to test the costs of begging in house sparrow (*Passer domesticus*) nestlings. In this way, we replicate two previous studies made on the same species [43], [44] by using basically the same experimental protocol: some nestlings are forced to beg for a long time (high begging group; hereafter HB) while others are fed shortly after they start begging (low begging group; hereafter LB). However, we have improved several aspects of the experimental methodology. For example (see Material and methods), we have used (i) larger sample sizes and (ii) a more complete experimental design. This allowed (iii) a paired approach in statistical analyses, which made it possible to control possible differences between nests. Also, we (iv) recorded more accurate data (i.e. by weighting each larva provided to the nestlings), (v) calculated a body-condition index instead with only the percentage of mass, (vi) applied a lower level of stress due to the organization of our aviary, and, mainly (vii) we performed a long-term experimental study (see Experimental Design).

Our main predictions are as follows:

First, with respect to immunocompetence, considering the clear results found in the three previously published papers testing this cost (see references above) we predict that nestlings from the HB treatment will present a lower immune response than nestlings from the LB treatment at both short-term and long-term levels (Prediction 1).

Second, two previous studies have failed to show delayed growth of house sparrow nestlings in relation to experimentally increased begging activity [43], [44]. However, considering that other studies have reported an effect of begging on growth in other species [42], [45], [47] and that several studies have demonstrated a trade-off between growth and immune response [66]–[68], we predict that an effect of begging on nestling condition should be found at least over the long term (Prediction 2).

Third, considering that mass loss triggered by begging (metabolic expenditure) showed a marginal difference between the HB and LB experimental groups in canaries (*Serinus canaria* [42]), although not in house sparrows [43], we predict that a long-term experimental study should find a significant effect of begging on metabolic expenditure (Prediction 3).

## Materials and Methods

### Ethics Statement

Research has been conducted according to relevant Spanish national (Real Decreto 1201/2005, de 10 de Octubre) and regional guidelines. All necessary permits were obtained from the Consejería de Medio Ambiente de la Junta de Andalucía, Spain. Approval for this study was not required according to Spanish law because it is not a laboratory study in which experimental animals have to be surgically manipulated and/or euthanatized.

In order to minimize intraspecific **competition**, food dishes (several of each type of food) were spaced throughout the aviary and there were more nest boxes than pairs (see below). Furthermore, nest-boxes were out of the aviary, in an adjacent laboratory, which allowed nest examination (and experimental manipulation, see below) from the laboratory.

### Study species, study population and general methods

The house sparrow is a colonial, very common, and broadly distributed passerine species [79] that, during the last years has become a model species for studies in evolutionary ecology given that it can be easily maintained in captivity, enabling more detailed and carefully controlled experimental studies [43], [44], [80]–[85].

This study was performed in a captive population of house sparrows maintained in an outdoor aviary of 375 m^3^ in the Faculty of Sciences (University of Granada, Spain). All sparrows were marked with a unique combination of colored rings, which allowed individual identification.

The birds were provided *ad libitum* access to commercial seed mix for canaries, nestling food for canaries with honey and small pieces of fruit added (egg food with fruits, manufactured by “Bogena”), cracked grains of wheat and rice, Diptera larvae and apple. Food dishes (several of each type of food) were spaced throughout the aviary to ensure that all birds had easy access to it. The aviary was provided with more nest boxes (*n* = 71) than pairs (*n* = 58 males and 59 females), and *ad libitum* access to vegetable material for nest construction was also provided during the breeding season. Nest-boxes were located in an adjacent laboratory and they were connected with the aviary across a tunnel of approximately 15 cm. We had access to the nest boxes from a different room to avoid having to disturb birds in the aviary. This allowed nest examination (and experimental manipulation, see below) from the laboratory, considerably decreasing potential stress to breeding birds. More detailed information on the aviary and sparrow care can be found in [86].

This study was carried out during the breeding season of 2012. From the beginning of the breeding season, nest-boxes were examined weekly, but when the construction of a nest was almost finished, the nest-box was checked daily, in order to collect precise information about laying date and clutch size. Pair members breeding in each nest-box were identified by observations or by video filming the nest entrance once the first egg was laid.

### Measurements and experimental design

Our long-term experimental treatment involved five consecutive days. Although long-term ecological studies usually extend more than five years, we think that this term can also be used in our study given that most studies on the cost of begging involve manipulation during a small portion of the nestling period while our manipulation covers a much larger period of development (i.e. five days). The experiment started when nestlings were 5 days old (hatching  = day 0), when house sparrow nestlings were growing at their highest rate [87]. Sparrows in our captive population laid between one and seven clutches (9 pairs with 1 clutch, 9 pairs with 2 clutches, 4 pairs with 3 clutches, 8 pairs with 4 clutches, 4 pairs with 5 clutches, 0 pairs with 6 clutches and 3 pairs with 7 clutches). In the experiment we used 4 first clutches, 10 second clutches, 6 third clutches and 1 fourth clutch. We did not find differences in the treatment effect depending on whether nestlings were from the first clutch or a subsequent one (results not shown), and thus this information was not included in the final analyses. Each pair was used only once.

Most experimental nests (19) contributed four nestlings to the experiment, one nest contributed three nestlings, and another nest contributed two nestlings. 17 non-experimental nests provided nestlings for substitution of experimental nestlings in their nests while our experiment was being performed. We used all chicks from the nest because this experimental design allowed us to control for possible differences between nests (see Statistical Analyses).

We took nestlings from the nests at 7:00 (local time) and replaced them with the same number of nestlings of the same age taken from non-experimental nests to avoid parental desertion. In the laboratory the nestlings were placed in artificial nests at a constant temperature of 28–32°C by putting an infrared lamp heater above the nestlings (during resting periods, nestlings were covered by a duster). Chicks were housed in pairs, i.e. the two chicks of the LB treatment together in an artificial nest and the two chicks of the HB treatment together in another one. The two artificial nests were located at about 10 m one from another in the lab so that the nestlings of one nest could not respond when we stimulated the nestlings of the other nest.

Experimental sessions started every day at 8:00 and ended at 20:30. Before the initiation and after the end of the experimental sessions we measured wing length and mass to the nearest 0.1 mm and 0.1 g (electronic balance Acculab, precision 0.01 g), respectively, which enabled us to calculate a body-condition index following Kedar et al. [44], as the residual from a lineal-regression line of mass over wing length. This body-condition index allowed us to control for the effect of size differences on mass differences, while percentage of initial mass (e.g. [43]) did not. To quantify the effects of begging on aspects of growth we have used body condition as a surrogate for growth throughout. Once weighed, nestlings were ranked according to their mass and alternately assigned to the high begging (HB) or low begging (LB) treatments. In this way, we created at most two pairs of HB – LB nestlings of similar mass within broods, one pair with the two largest nestlings (nestling rank large) and another pair with the two smallest nestlings (nestling rank small). We alternated the order of assignment of the HB and LB treatments between consecutive nests; thus, the heaviest nestling was assigned to the HB treatment in half of the broods and to the LB treatment in the other half.

During the first day of the experiment, following Moreno-Rueda [43], nestlings were fed with one Diptera larva every 30 min. However, during the subsequent days they were fed every 20 min and the number of larvae was increased to match the increase of nestling mass. Thus, all experimental nestlings received exactly the same number of larvae every day, a quantity that matches the number of larvae provided by parents in the aviary [43; personal observations].

Our experimental treatment consisted of stimulating chicks from the HB treatment to beg for longer and chicks from LB treatment for shorter periods than under natural conditions, which is the usual protocol used in these type of studies [24], [42]–[45], [47], [83]. Following Moreno-Rueda [43], we stimulated nestlings from the HB treatment to beg for 60 s every 10 min. Begging stimulation was made by whistling and dangling a larva close to the chicks' bills. Each third period of stimulation (i.e. every 30 min) the nestlings were fed at the end of the 60 s of begging. Nestlings from the LB treatment were stimulated to beg only once every 30 min, just when they had to be fed, and we provided them with the larva as soon as they gaped; thus, they never begged for more than a few seconds. This means that nestlings in the HB treatment begged for a total of 360 s per hour while nestlings in the LB treatment begged in all cases for less than 10 s per hour.

The immunological costs of begging were determined by measuring *in vivo* cell-mediated immune response following standardized protocols in previously published papers, mainly those using the house sparrow as model species [43], [84], [88]. The first day of the experiment, before the start of the first feeding trial, we injected subcutaneously 0.1 mg of an antigen (phytohaemagglutinin; PHA-P, L-8754; Sigma Aldrich) dissolved in 0.02 ml of physiological saline solution (Bausch & Lomb Co.) in the left wing web. The right wing web was injected with 0.02 ml of saline solution and thus used as control. Later on, the fourth day of the experiment, in order to test for the long-term effect of begging on the immune response, we repeated the immunological test by injecting 0.03 ml of the same solution of PHA (the increase in volume of the injected solution was to match the mass increase of nestlings) on the right wing web, while the saline solution (i.e. control) was injected in the left wing web. The injection of this antigen acts as an infection and provokes an inflammatory immune reaction, which provides effective protection against infections triggered by bacteria and viruses [89]–[91]. We measured the thickness of each wing web (i.e. the skin between humerus and ulna-radius bones) at the injection site with a digital pressure-sensitive micrometer (Mitutoyo, model ID-CI012 BS, precision 0.01 mm) before and 6 hours after the injection the first day of the experiment (following [43], i.e. short-term effect of the immunological costs of begging, hereafter pha-1). Moreover, we also measured the thickness of each wing web at the end of the first day and at the beginning and at the end of each subsequent days of the experiment (i.e. long-term effect of the immunological costs of begging, hereafter pha-2). In all cases, we repeatedly measured each wing web three times and, since they were highly repeatable (one-way ANOVAs, left wing: *F*_848,1697_ = 358.48; *P*<0.00001, Adjusted-R^2^ = 0.99; right wing: *F*_848,1697_ = 1545.1; *P*<0.00001, Adjusted-R^2^ = 1.00), the mean value was used in subsequent analyses (see e.g. [66], [84]). As the degree of swelling is considered an indication of the strength of the immune response [66], [92], we calculated the PHA response (i.e. wing web index) as the change in thickness of the experimental wing web (i.e. the one injected with PHA) minus the change in thickness of the control wing web (the one injected with saline solution).

To determine the metabolic costs for both HB and LB nestlings, following Kilner [42], we calculated, for each nestling (i) the exact amount of food ingested (each larva was weighted individually just before given to the nestlings), (ii) mass gained during each experimental session (i.e. final mass minus initial mass), and (iii) exact mass excreted (i.e. all fecal sacs from each nestling were collected soon after excretion and immediately weighed). The mass of larvae and fecal sacs were measured to the nearest 0.01g (electronic balance Acculab, see above). Metabolic costs were calculated by subtracting mass gained and mass excreted from the mass ingested.

### Statistical procedures

We tested the effect of our treatment on nestling immune response, nestling-body condition (residual nestling weight against wing length), increment in body weight (mass gained) and metabolic cost (larva weight – feces weight – increment in body weight). Moreover, we also analyzed whether there was an effect of our manipulation on mass excreted (i.e. feces weight) in order to rule out any confounding effects explaining our results. For data analysis, of we used mainly Linear Mixed Models performed in R v2.15.3 [93] by using *nlme* (R package v.3.1–108 [94]). As random effects, we used nest identity and nestling identity (nested in nest identity) as two random factors. As fixed effects, we included treatment (HB vs. LB) as a fixed factor, time (in hours) after the beginning of the experiment for each brood (hereafter time) as fixed continuous predictor, and the interaction between time and treatment. For the analyses of nestling immune response, we used time (in hours) from the last injection instead of time after the begging of the experiment. We also included the immune trial number (pha-1 or pha-2) as a further factor (hereafter pha-trial), and their interactions with time from injection and treatment, respectively (only second-order interactions).

We have followed Diggle et al. [95] and Zuur et al. [96] to perform the model selection. Firstly, we estimated the best structure for random effects by including all fixed effects and their interactions in the model and then comparing different models with an increased complexity in random structure (no random effect, only random intercept, and random intercept and random slopes, including step by step the slope of each fixed component and afterwards the slopes of their interactions). These nested models were adjusted by REML and compared by the ANOVA function.

Once the best structure of random effects was determined, we made similar analyses to select the best structure for the fixed effects. We compared successive models with an increasing number of fixed components, from no fixed effects to the full model and by using the best random structure previously determined; in this case, we used ML to adjust the statistical models. After determining the best structures for both fixed and random effects, we fixed the final model by REML and checked the model assumptions, i.e. no pattern when plotting residual *vs*. Predicted values, no pattern when plotting residual vs. predictors, and normality of model residuals. We calculated the effect sizes the linear mixed models following Nakagawa and Schielzeth [97] and Johnson [98] and by using the code in R available from http://jonlefcheck.net/2013/03/13/r2-for-linear-mixed-effects-models. Two values are reported: the marginal R2 (*R^2^_GLMM(m)_*) that describes the proportion of variance explained by the fixed factor(s) alone; and the conditional R^2^ (*R^2^_GLMM(c)_*), which describes the proportion of variance explained by both the fixed and random factors.

Additionally, we tested the effect of our experimental treatment in each successive experimental session performed during nestling development. For these analyses, we used a design similar to that used by Moreno-Rueda [43] but with some improvements due to our paired design (i.e. HB and LB treatments were applied in siblings from the same nest). Namely, we used repeated measures analyses for variance (RM-ANOVA) performed in STATISTICA v.8 (StatSoft 2008). In this way, we could test differences in the dependent variables among nestlings from the same nests, and thus control for possible differences between nests. In short, we used RM-ANOVA with nestling rank (large vs. small) and experimental treatment (HB vs. LB) as two within-factors. We also included the interaction factor between within-factors, in order to examine whether the effect of the treatment varied with nestling rank. Moreover, we used LSD *post hoc* tests to determine separately the effects of the experimental treatment in small and large nestlings. We have also calculated the effect sizes (partial eta-square) for these analyses. Nonetheless, this design does not allow missing data, and thus only those cases with measurements from the four nestlings could be included in these analyses.

## Results

### Initial conditions

At the beginning of the experiment (time 0), nestlings assigned to higher rank were significantly heavier (F1, 18 = 114.42, P<0.00001, Table 1), had longer wings (F1, 18 = 43.42, P<0.00001) and thus showed a better body condition (F1, 18 = 50.89, P<0.00001) than nestling assigned to lower rank (Table 1). Notably, there were not significant differences for these three variables at this initial time between siblings assigned to different treatments (weight: *F*_1, 18_ = 0.34, *P* = 0.57; wing: *F*_1, 18_ = 0.38, *P* = 0.85; body condition: *F*_1, 18_ = 1.07, *P* = 0.31, Table 1), nor any significant interaction between treatment and nestling rank for these variables (weight: *F*_1, 18_ = 0.32, *P* = 0.58; wing: *F*_1, 18_ = 2.15, *P* = 0.16; body condition: *F*_1, 18_ = 0.35, *P* = 0.56, Table 1).

**Table 1:** Corporal measurements (LSmeans (−95%CI±95% CI)) at the beginning of experiment (time  = 0). N = 19 nests, 76 nestlings.

Nestlings Rank	treatment	Weight (g)	Wing (mm)	Body condition
Large	HB	9.96 (9.37–10.56)	17.63 (18.85–0.83)	0.83 (0.36–1.30)
Large	LB	9.90 (9.22–10.59)	18.39 (19.54–0.54)	0.54 (−0.04–1.12)
Small	HB	7.94 (7.37–8.51)	14.97 (16.44– −0.37)	−0.37 (−0.81–0.07)
Small	LB	7.68 (7.07–8.29)	14.42 (15.77– −0.46)	−0.46 (−0.93–0.01)

### Immunological costs

**Figure 1:**
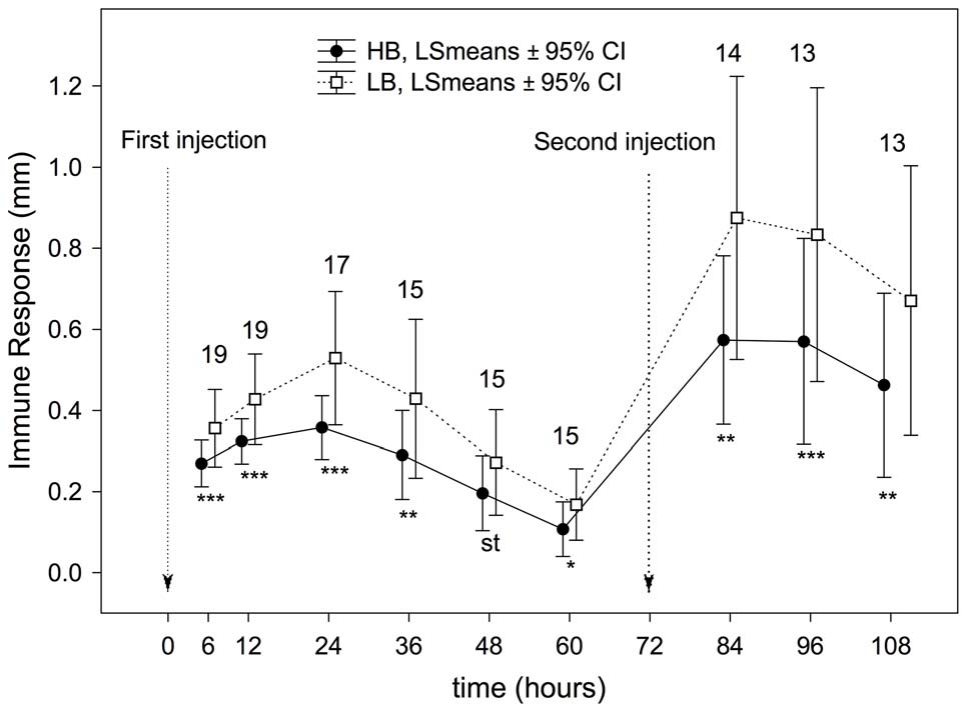
Effect of the experimental treatment on immune response calculated from RM-ANOVAs performed for each experimental session. P-values associated with differences in each experimental session are indicated as ns: *P*>0.05; st: 0.1≤*P*≥0.05; *: *P*<0.05; **: *P*<0.001 and ***: *P*<0.0001. Numbers of nests (i.e. those with measurements from the four nestlings) used in each of the comparisons are also shown.

**Figure 2:**
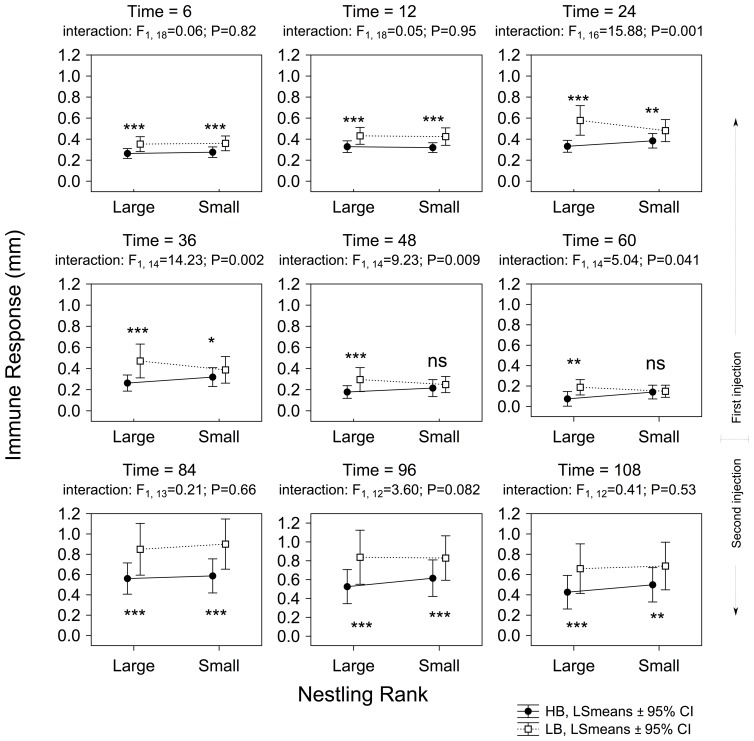
Effects of the experimental treatment on the immune response according to nestling rank calculated from RM-ANOVAs in the successive experimental sessions. The significance of interactions between experimental treatment and nestling rank are also shown. P-values associated to LSD *post hoc* tests (i.e. treatment effect within nestling rank) are indicated as ns: *P*>0.05; st: 0.1≤*P*≥0.05; *: *P*<0.05; **: *P*<0.001 and ***: *P*<0.0001.

The greater begging effort made by HB nestlings provoked a higher immune response in these nestlings compared to LB nestlings, even at the first measurement (6 h, Fig. 1). This clear effect of experimental treatment on the immune response persisted, regardless of the time at which the response was measured (Fig. 1). However, the effect of our experimental treatment was not similar for large and small nestlings during pha-1, as interactions between treatment effect and nestling rank resulted significant from time 24 h until last measure before pha-2 (i.e. time  = 60 h, see Fig. 2). In all these cases, the interaction reached significance because the significant effects of treatment on immune response in small nestlings decreased quicker than in large nestlings. In short, from time  = 48 h, no effect was found in small nestlings (see Fig. 2 and Table S1). These differences between small and large nestlings were not found during pha-2, because the effect of experimental nestling was maintained in a similar way in small and large nestlings (see Fig. 2 and Table S1).

When all immune response measurements were considered, nestlings from the HB treatment presented a lower immune response than did nestlings from the LB treatment (treatment effect, Table 2A). The immune response was higher during the second test (pha-2) than during the first test (pha-1) (trial-pha effect, Table 2A). Moreover, the interaction between treatment and pha-trial proved significant (Table 2A), indicating that differences of immune response between treatments were higher during the second test than during the first test (Fig. 1).

**Table 2:** Final models from analyses (Linear Mixed Models fixed by REML) using measurements from all experimental sessions.

A) Inmunological costs					
Random effects: nestling nested in nest					
random intercepts and random slopes (treatment + pha_trial)					
Fixed effects:	Value	Std.Error	df	t-value	p-value
(Intercept)	0.48	0.04	609	13.17	<0.00001
Treatment	−0.12	0.02	59	−4.77	<0.00001
pha_trial	0.45	0.05	609	8.29	<0.00001
Time	<0.01	<0.01	609	−12.09	<0.00001
Treatment × pha_trial	−0.17	0.03	609	−5.93	<0.00001
Number of Groups: 21 nests and 81 nestlings. *R^2^_GLMM(m)_* = 0.28; *R^2^_GLMM(c)_* = 0.89					

B) Body condition costs					
Random effects: nestling nested in nest					
random intercepts and random slopes (treatment + time)					
Fixed effects:	Value	Std.Error	df	t-value	p-value
(Intercept)	0.19	0.18	766	1.02	0.31
Treatment	0.06	0.19	59	0.31	0.76
Time	<0.01	<0.01	766	−0.38	0.71
Treatment × time	−0.01	<0.01	766	−3.65	0.0003
Number of Groups: 21 nests, 81 nestlings. *R^2^_GLMM(m)_* = 0.03; *R^2^_GLMM(c)_* = 0.80					

C) Mass gained					
Random structure: nests					
random intercepts and random slopes (treatment + time)					
Fixed effects:	Value	Std.Error	df	t-value	p-value
(Intercept)	0.62	0.06	441	11.30	<0.00001
treatment	−0.17	0.06	441	−2.79	0.0056
Time	0.01	<0.01	441	13.70	<0.00001
Number of Groups: 21 nests, 81 nestlings. *R^2^_GLMM(m)_* = 0.43; *R^2^_GLMM(c)_* = 0.52					

D) Mass excreted					
Random effects: nests					
random intercepts					
Fixed effects:	Value	Std.Error	df	t-value	p-value
(Intercept)	0.27	0.02	441	12.76	<0.00001
treatment	−0.02	0.02	441	−1.01	0.31
Time	0.01	<0.01	441	28.47	<0.00001
Number of Groups: 21 nests. *R^2^_GLMM(m)_* = 0.62; *R^2^_GLMM(c)_* = 0.65					

E) Metabolic costs					
Random structure: nests					
random intercepts and random slopes (treatment + time + time: treatment)					
Fixed effects:	Value	Std.Error	df	t-value	p-value
(Intercept)	0.29	0.04	423	6.95	<0.00001
treatment	0.14	0.05	423	2.78	0.0057
Time	0.01	<0.01	423	7.51	<0.00001
Number of Groups: 21 nests. *R^2^_GLMM(m)_* = 0.26; *R^2^_GLMM(c)_* = 0.32					

The marginal R^2^ (*R^2^_GLMM(m)_*), and the cnditional R^2^ *(R^2^_GLMM(c)_*) for each model are also shown (see methods).

### Body condition costs

**Figure 3:**
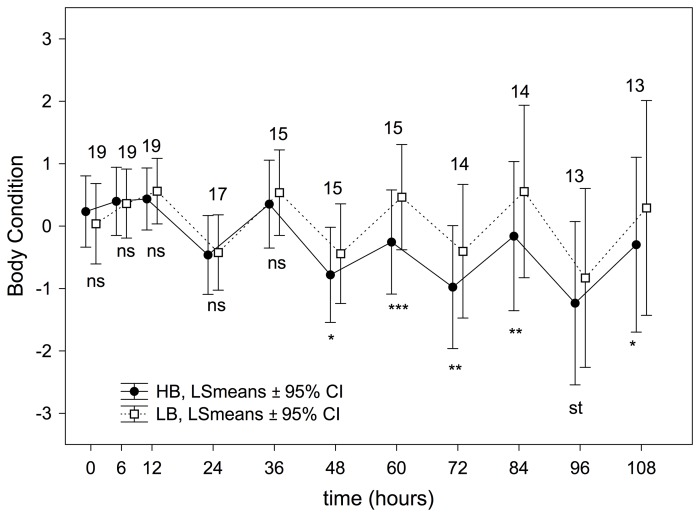
Effect of the experimental treatment on body condition calculated from RM-ANOVAs over nestling development. P-values associated with differences in each experimental session are indicated as ns: *P*>0.05; st: 0.1≤*P*≥0.05; *: *P*<0.05; **: *P*<0.001 and ***: *P*<0.0001. Numbers of nests (i.e. those with measurements from the four nestlings) used in each comparisons are also shown.

During the first day and a half of the experiment, the treatment had no effect on body condition (6 to 36 hours, Fig. 3). However, after 48 h HB nestlings showed a lower body condition than did LB nestlings (Fig. 3), a result maintained during the subsequent measurements (Fig. 3). Moreover, the detected effect of treatment did not differ depending on nestling rank (i.e. no significant interactions between treatment and nestling ranks; see Table S2). Overall, the interaction between treatment and time was significant (Table 2B), because the body condition of HB nestlings decreased as the treatment time increased, an effect not found in LB nestlings (Fig. 3).

### Mass gained

**Figure 4:**
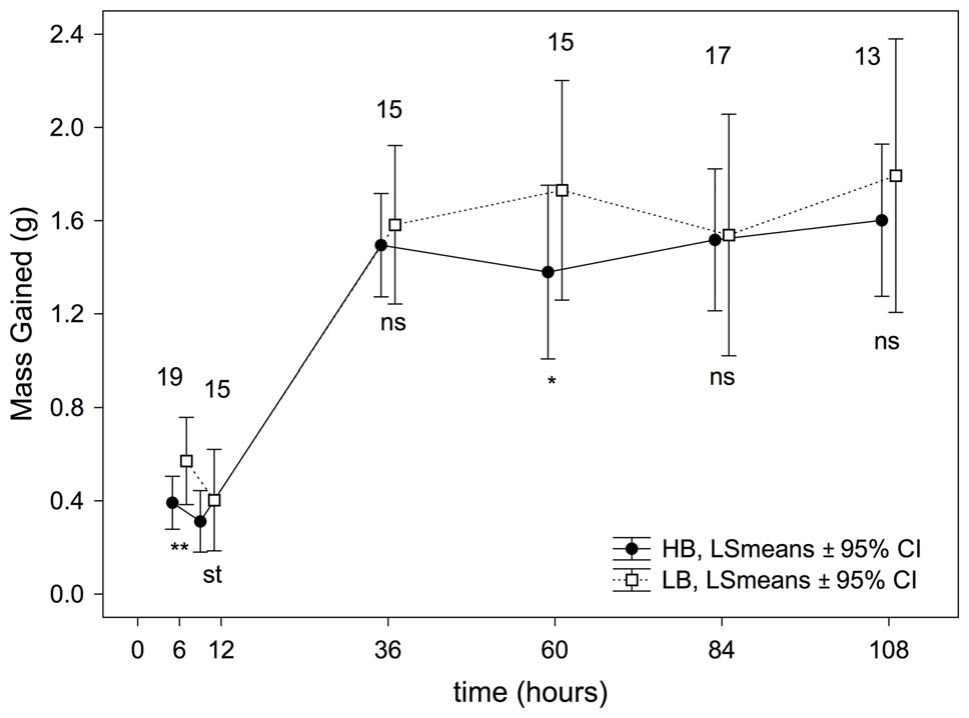
Effect of the experimental treatment on mass gained by nestlings and calculated from RM-ANOVAs in each experimental session. P-values are indicated as ns: *P*>0.05; st: 0.1≤*P*≥0.05; *: *P*<0.05; **: *P*<0.001 and ***: *P*<0.0001. Numbers of nests (i.e. those with measurements from the four nestlings) used in each one of comparisons are also shown.

The experimental treatment significantly affected the mass gained during experimental sessions. In short, HB nestlings gained less mass than LB nestlings did (treatment effect, Table 2C). This effect was also found on separately analyzing each experimental session for time 6, time 12, and time 60 (see Fig. 4).

### Mass excreted

**Figure 5:**
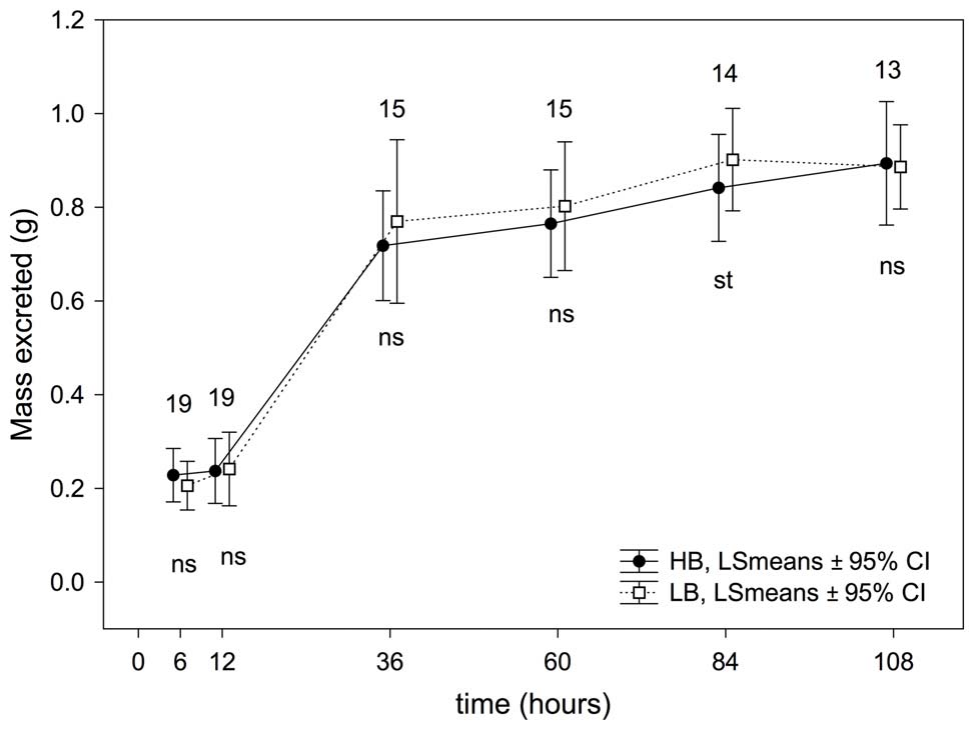
Effect of the experimental treatment on mass excreted by nestlings based on the RM-ANOVAs for each experimental session. P-values associated to LSD *post hoc* test are indicated as ns: *P*>0.05; st: 0.1≤*P*≥0.05; *: *P*<0.05; **: *P*<0.001 and ***: *P*<0.0001. Numbers of nests (i.e. those with measurements from the four nestlings) used in each comparison are also shown.

**Figure 6:**
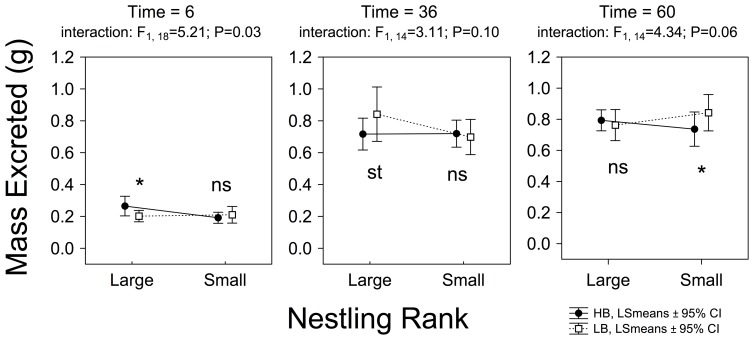
Effects of the experimental treatment on mass excreted by nestlings according to nestling rank calculated from RM-ANOVAs in the experimental sessions where the interaction effects between treatment and nestling rank resulted significant (or almost). The significance values of interactions between experimental treatment and nestling rank are also shown. P-values associated to LSD *post hoc* (i.e. treatment effect within nestling rank) tests are indicated as ns: *P*>0.05; st: 0.1≤*P*≥0.05; *: *P*<0.05; **: *P*<0.001 and ***: *P*<0.0001.

We found no overall effect of the experimental treatment on the mass excreted by nestlings (see Table 2D and Fig. 5). We found one significant result and two trends for the interaction effects between treatment and nestling rank in time 6, time 36, and time 60, respectively (see Fig. 6). Namely, in time 6, HB-large nestling excreted more than LB-large nestlings, but the opposite happened in time 36; in time 60, HB-small nestlings excreted less mass than LB-small nestlings (see Fig. 6).

### Metabolic costs

**Figure 7:**
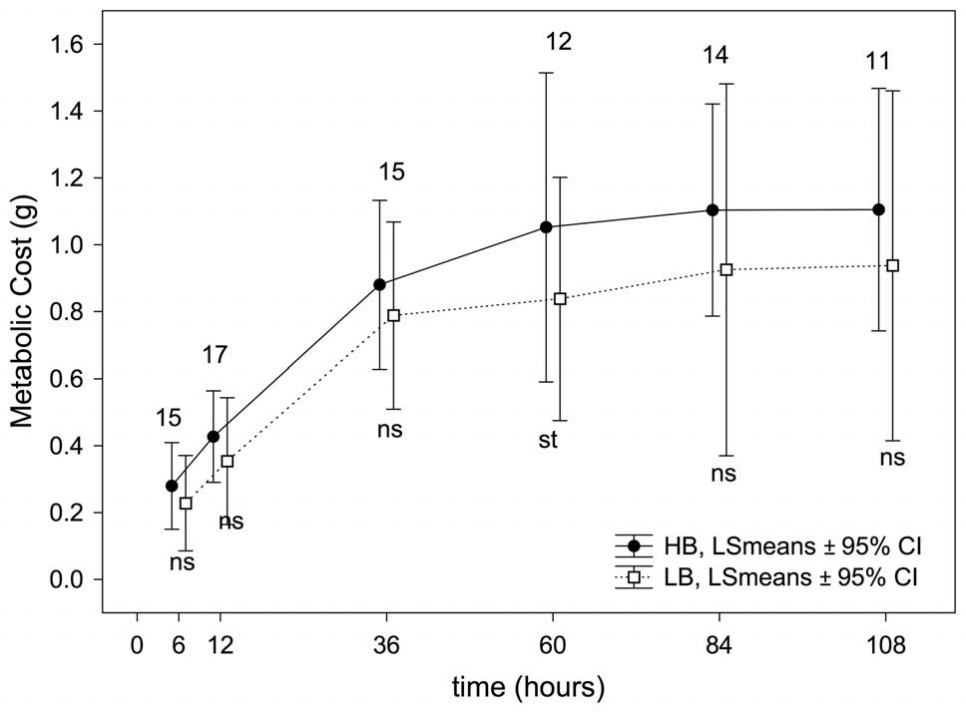
Effect of the experimental treatment on metabolic costs of nestlings based on the RM-ANOVAs performed in each experimental session. P-values are indicated as ns: *P*>0.05; st: 0.1≤*P*≥0.05; *: *P*<0.05; **: *P*<0.001 and ***: *P*<0.0001. Numbers of nests (i.e. those with measurements from the four nestlings) used in each comparison are also shown.

Overall, the experimental treatment significantly affected the metabolic expenditure, this being higher in HB nestlings than in LB nestlings (treatment effect, Table 2E, Fig. 7). However, this effect did not reach statistical significance (only a statistical trend in time 60 h, P = 0.054, see Table S3) when the treatment effect was analyzed separately for each experimental session (Fig. 7).

## Discussion

Some short-term experimental studies have tested the hypothesized physiological costs of usually vigorous and exuberant begging signals, but this subject remains elusive given that published results frequently fail to support the presumed costliness of begging and are often contradictory (see Introduction). This inconsistency between empirical data and predictions derived from models of costly begging could be, at least partially, because these studies experimentally manipulate begging behaviour over a short time period (usually less than one day). Nestlings beg for food over the entire nestling period, and perhaps one day of experimental manipulation is enough only to detect the full costs in each species. Here we examine this scenario in a long-term experimental study in which we manipulated the duration of begging displays of house sparrow nestlings over five days of experimental treatment.

The resources used by nestlings in costly begging behaviour could be diverted from physiological processes, mainly immune response, growth, and metabolism. We have found that HB nestlings mounted a smaller immune response to phytohaemagglutinin than did LB nestlings (Fig. 1), confirming the existence of an immunological cost of begging, as demonstrated in three previous experimental studies [43], [47], [58]. Thus, it can be considered well documented that experimentally increased levels of begging provoke costs in terms of immunocompetence, which could have drastic consequences because nestlings with reduced immune capacity have a higher mortality risk [55]–[57]. Furthermore, nestlings from the HB treatment presented a lower immune response than did nestlings from the LB treatment, even since the first measurement (6 h), and this clear effect was maintained in all the immune-response measurements (Fig. 1). It bears mentioning that the effect of the treatment was higher during the second than during the first test (Fig. 1, Table 2A), indicating that the immunological cost of begging is clearer over the long term than the short.

Another noteworthy result highlighted by our long-term experimental treatment is that large and small nestlings responded differently to the experimental treatment during pha-1: large nestlings maintained their immunological response during all measurements (time 60 h), while in small nestlings this response disappeared at time 48 h and time 60 h. This is presumably the consequence of smaller nestlings not being able to maintain the costly immunological response for such a long time. Notably, this difference between large and small nestlings disappears when they are older, in the second test (pha-2), suggesting a potential effect of age in how nestlings handle begging costs.

The existence of a growth cost of begging is less clear in some species than others (see Introduction). Specifically, in the house sparrow, two experimental studies have failed to show delayed growth of nestling forced to beg longer [43], [44]. However, our long-term experiment has shown that the body condition of HB nestlings started to worsen after 48 h of treatment, and this deterioration intensified as the treatment time lengthened (Fig. 3, Table 2B). Thus, our study contradicts the absence of trade-off between growth and begging in the house sparrow found in the two previously short-term experimental studies cited above. Our results suggest that a growth cost of begging could likely be found in long-term experiments in most species.

The experimental treatment significantly affected the mass gained during experimental sessions. HB nestlings gained less mass than LB nestlings (Table 2C, Fig. 4). However, the experimental treatment did not have any effect on the mass excreted by nestlings (see Table 2D and Fig. 5). This result is important because Kilner [42] found that HB nestlings produced a greater number of fecal sacs than did LB nestlings, implying that begging could indirectly exert a growth cost by affecting digestive efficiency [42]. This idea was later suggested in several papers [14], [99]. Our results showing that mass excreted by HB nestlings was similar to that excreted by LB nestlings do not support the existence of the purported digestive costs of begging, confirming results from more recent published papers [82], [83], [100].

The effect of high begging on metabolic expenditure has proved less clear. We found no significant differences when analyzing the effect of treatment separately for each experimental session; however, overall, the long-term metabolic expenditure was higher in HB than in LB nestlings (Table 2C, Fig. 7).

This is the first study to show such a substantial metabolic cost of begging, given that Kilner [42] reported only a marginal difference between experimental groups in canaries. Moreno-Rueda [58] found no effect of begging on metabolic expenditure in house sparrows. Our study is also the first to demonstrate a significant cost of begging with respect to three physiological processes: immunocompetence, growth, and metabolic expenditure.

One controversial point is whether or not different species are specialized in re-allocating resources for begging displays from different physiological functions, as suggested by Moreno-Rueda [58]. This suggestion seems unlikely, because (i) physiological processes such as immune response, growth, and metabolism are all costly [52]–[54], [101], [102]; (ii) it is well known that during development there is a trade-off in resource utilization between growth and other physiological functions (66–68, 101, 103–105), given that energy and nutrients required for growth are often limited [52], [101]; and (iii) investment in different physiological functions should be adjusted according to the availability of resources and ecological conditions [52], [69], [103]–[106]. Therefore, all physiological costs likely occur simultaneously -that is, a multilevel trade-off occurs between begging and all physiological costs. Supporting this statement, Moreno-Rueda and Redondo [47] reported that high levels of begging provokes both immunoresponse and growth costs in southern shrike (*Lanius meridionalis*) nestlings, and, mainly, our long-term experimental study has shown a significant effect of begging on three different physiological processes.

One of the arguments used to support the idea of the species-specific cost of begging was that in species in which a growth cost of begging had not been detected, the energy needed for begging would have been diverted from the immune system [43]. This suggestion is also unlikely because, as specified above, differential investment in physiological functions is driven by availability of resources and ecological conditions. This means that developing nestlings should only dedicate comparatively more valuable resources to their immune system when the associated benefits are higher, i.e. when the risk of being infected is high [52], [107], [108]. Thus, if the risk of infections is very low, investment in the immune system would be very low as well, and thus no energy could be diverted from the immune system. A multilevel trade-off between begging and physiological costs is probably mediated by steroid hormones and by oxidative stress. The effect of hormones has been clearly documented [50], [51], [109]–[112], although the role of oxidative stress remains to be clearly demonstrated. Begging is presumably an antioxidant demanding activity that entails production of reactive molecular species, which can produce oxidative damage at different levels because (i) it has been shown that begging intensity negatively covaried with oxidative damage [113]; and (ii) it has been demonstrated that when nestlings are administered vitamin E, a non-enzymatic antioxidant, some components of begging displays were enhanced [114].

In conclusion, our long-term experiment in the house sparrow has provided evidence of a growth cost of begging that two previous studies failed to show in this same species. This is the first study to demonstrate a metabolic cost of begging, and also the first to show a significant cost of begging with respect to three physiological processes simultaneously: immunocompetence, growth, and metabolic expenditure.

## Supporting Information

Data S1 **Data file including all data used in the study.**
(XLS)

Table S1 **Results for nestling Immune Response in each experimental sessions.** Analyses are RM-ANOVAs with nestling rank (large vs small) and experimental treatment (HB vs LB) as two within factor.
(XLS)

Table S2 **Results for nestling body Condition in each experimental sessions.** Analyses are RM-ANOVAs with nestling rank (large vs small) and experimental treatment (HB vs LB) as two within factor.
(XLS)

Table S3 **Results for nestling Metabolic Costs in the experimental sessions.** Analyses are RM-ANOVAs with nestling rank (large vs small) and experimental treatment (HB vs LB) as two within factor.
(XLS)
